# Author Correction: Electro-capillary peeling of thin films

**DOI:** 10.1038/s41467-024-46369-7

**Published:** 2024-03-04

**Authors:** Peiliu Li, Xianfu Huang, Ya-Pu Zhao

**Affiliations:** 1 State Key Laboratory of Nonlinear Mechanics, Institute of Mechanics, Chinese Academy of Sciences, Beijing, China; 2 School of Engineering Science, University of Chinese Academy of Sciences, Beijing, China

**Keywords:** Engineering, Mechanical engineering, Wetting

Correction to: *Nature Communications* 10.1038/s41467-023-41922-2, published online 03 October 2023

1. The original version of this article contained an error at two locations (12^th^ line of 4^th^ paragraph of the introduction, 4^th^ line of 10^th^ paragraph of results section “Theoretical model and dynamic analysis of the electro-capillary peeling”) (corresponding to the text of “described by *r* ∼*U*, *r* ∼*d*_0_^‒1/2^ and *r* ∼*E*^‒1/2^”), where the power exponent of thickness *d*_0_ and elastic modulus *E* incorrectly reads ‒1/2. The correct power exponent should be ‒2. The correct text is “*r* ∼*U*, *r* ∼*d*_0_^‒2^ and *r* ∼*E*^‒2^”.
2. The legend of Fig. 4e, incorrectly have power exponent of thickness *d*_0_ and elastic modulus *E* as ‒1/2. The correct power exponent should be ‒2. The correct text is “*r*(*t*) ∼*d*_0_^‒2^, and *r*(*t*) ∼*E*^‒2^”.
3. The original version of this Article contained an error in Fig. 4e, where the annotation line of the power exponent is incorrectly given 1:2. The corrected ratio is 2:1. The correct version of Fig. 4 is:

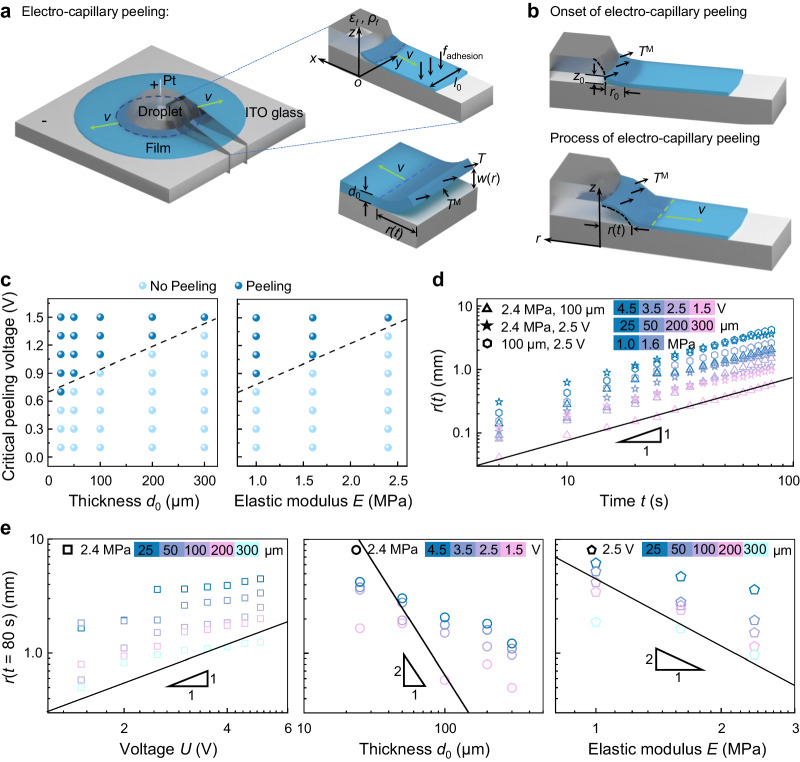

  which replaces the previous incorrect version:

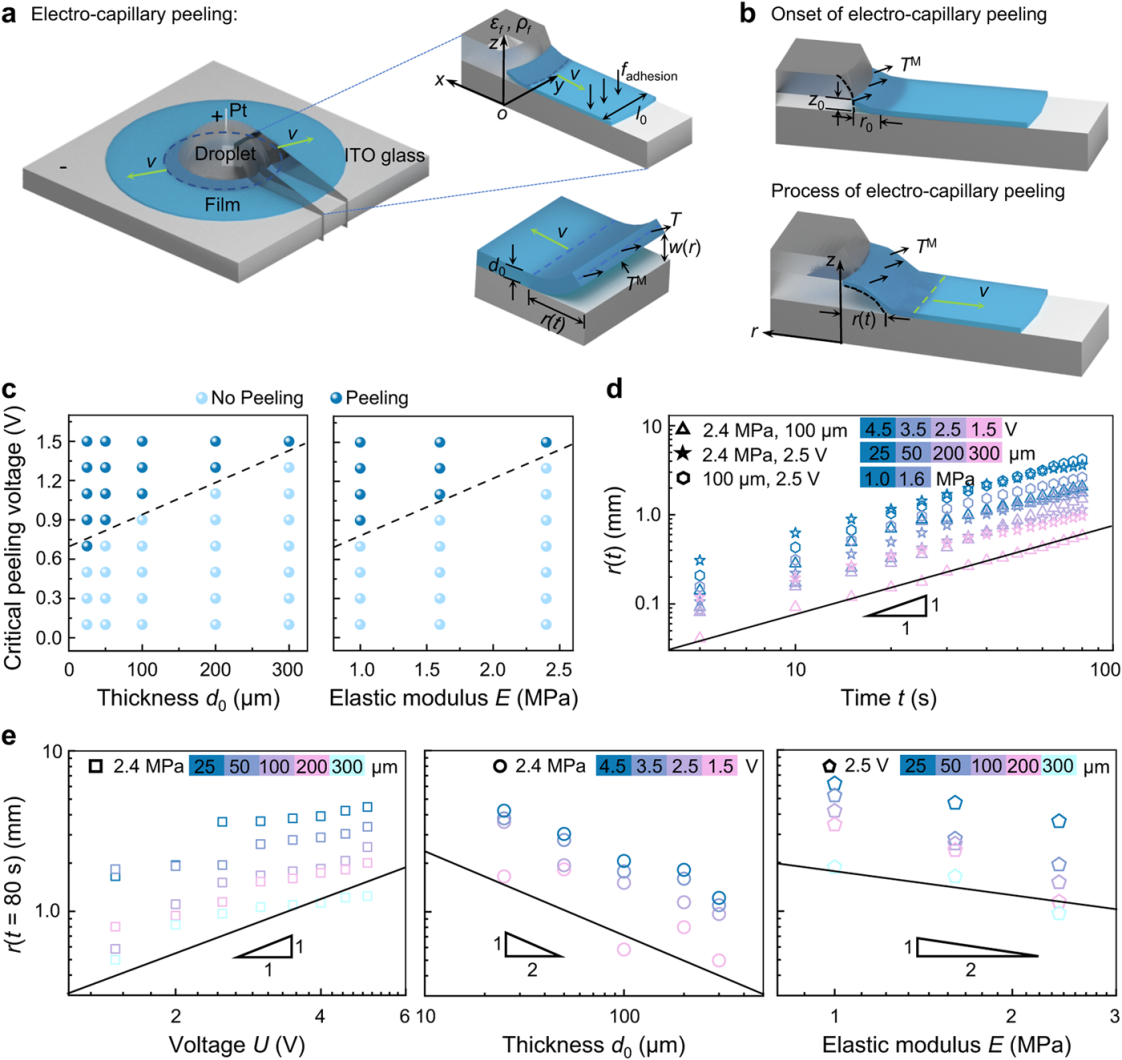

4. The original version of this article contained an error at 8^th^ line of 10^th^ paragraph of results section “Theoretical model and dynamic analysis of the electro-capillary peeling” that incorrectly reads “The peeling length is proportional to the peeling time, and the impact of the applied voltage and film properties on the electro-capillary peeling are all consistent with the analysis. Within the uncertainties, excellent agreement is shown between the predicted and experimental data.” Considering that the thin film used in the experiment is not an ideal film, and its bending stiffness exists and is affected by the thickness and elastic modulus. The correct text should read as, “The peeling time and applied voltage, the relationship between them and the peeling length is consistent with the analysis due to the bending stiffness’s assumption having no effect. Within the uncertainties, an agreement is shown between the predicted and experimental data on the relationship between peeling length and peeling time/applied voltage, and the working mechanism describes the trend of the influence of thin film thickness and elastic modulus on the peeling length.”

The errors have been corrected in HTML and PDF versions of the article.

