# Supplementary material for: Electro-capillary peeling of thin films
Source: Nat Commun. 2023 Oct 3;14:6150. doi: 10.1038/s41467-023-41922-2 (PMC10547721; doi:10.1038/s41467-023-41922-2)
Supplement: Supplementary file 1 — Supplementary Information [file 41467_2023_41922_MOESM1_ESM.pdf]

Supplementary Information

Electro-capillary peeling of thin films

Peiliu Li[1,2], Xianfu Huang[1,2] & Ya-Pu Zhao[1,2]✉

[1]State Key Laboratory of Nonlinear Mechanics, Institute of Mechanics, Chinese Academy of Sciences, Beijing, China.

[2]School of Engineering Science, University of Chinese Academy of Sciences, Beijing, China.

✉Corresponding author: yzhao@imech.ac.cn

The PDF file includes:

Supplementary Figs. 1 to 14

Supplementary Tables 1 to 4

Other Supplementary Material for this manuscript includes the following:

Supplementary Movies 1 to 4

Supplementary Figures

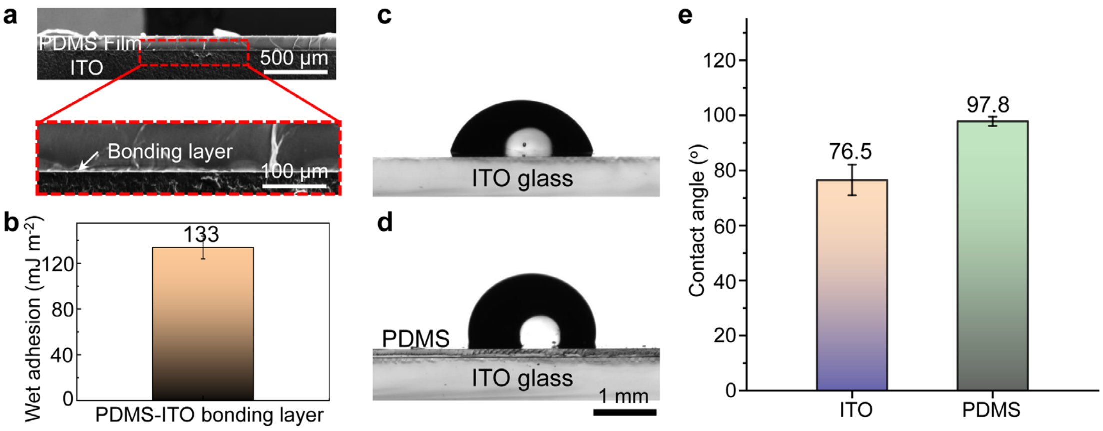

Supplementary Fig. 1. The interfacial features in the electro-capillary peeling method. a SEM images of the PDMS film on the ITO glass. The bonding layer is tight without any microchannels. **b** Wet adhesion of the bonding layer. The wet adhesion of the bonding layer is about 133 mJ m^{-2}. **c-e** Wettability of ITO glass surface and PDMS film. The liquid droplet is tested with a volume of 10 μL, and the contact angle of the ITO glass and PDMS surface are about 76.5° and 97.8°, respectively. The error bars in **e** are the standard deviation of the raw data.

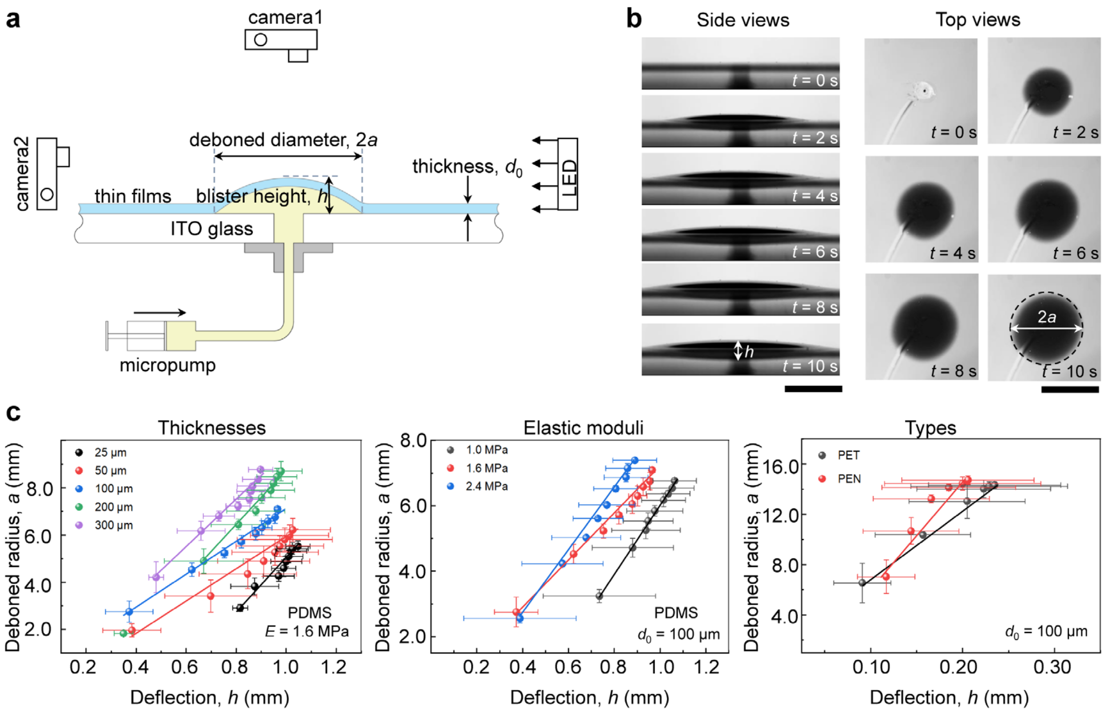

Supplementary Fig. 2. Water blister measurement of the bonding layer wet adhesion. a Schematic illustration of the water blister measurement. Dyed water is injected through the inlet underneath the thin film. The side-view and top-view images of the blister are captured to determine the maximum deflection h and debonded diameter $2a$. **b** Representative image of the delamination front during a blistering test. The debonded radius (a) is estimated by fitting a curve to the edge of the region where the dyed fluid is visible. **c** Experimental results of aqueous injection. The debonded radius and deflection are tested with a thin film of various thicknesses, elastic moduli, and types. Debonded radius versus deflection to determine the interface wet adhesion. The error bars in **c** are the standard deviation of the raw data.

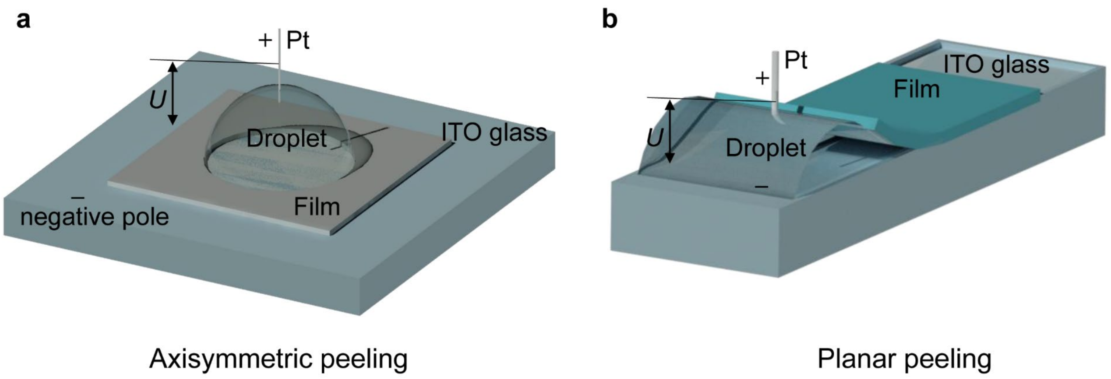

Supplementary Fig. 3. Experimental setup of axisymmetric and planar peeling. a Axisymmetric peeling. A circular hole is prefabricated on the PDMS films, and an electrolyte droplet is placed into this circular hole. **b** Planar peeling. There is a closed frame for wrapping the PDMS films.

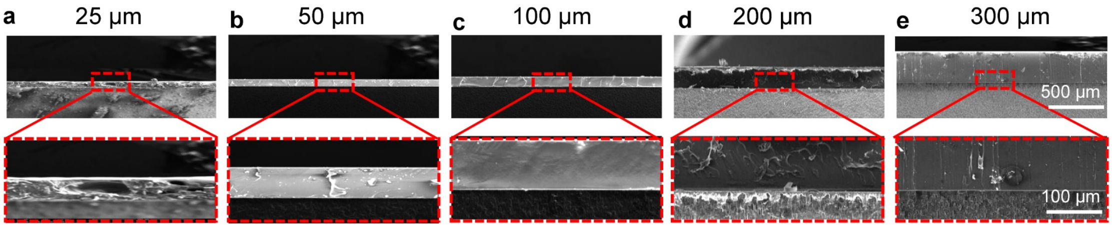

Supplementary Fig. 4. SEM images of PDMS films with various thicknesses on the ITO glass surface. The thicknesses of **a-e** are 25, 50, 100, 200, and 300 µm, respectively. The red dotted line box is a partially enlarged view of the bonding layer. Scale bars of images in the top row are 500 µm, and in the bottom row are 100 µm.

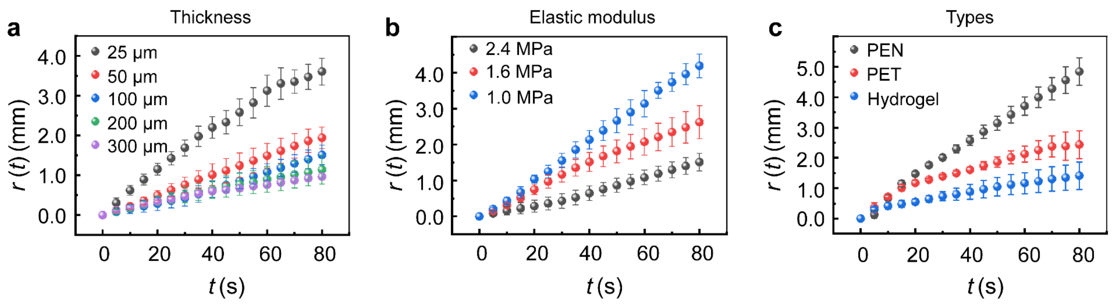

Supplementary Fig. 5. Statistical results of peeling length. a-c The evolution of peeling length for the film with various thicknesses, elastic moduli, and types. All experiments were tested at a voltage of 2.5 V. The error bars in **a-c** are the standard deviation of the raw data.

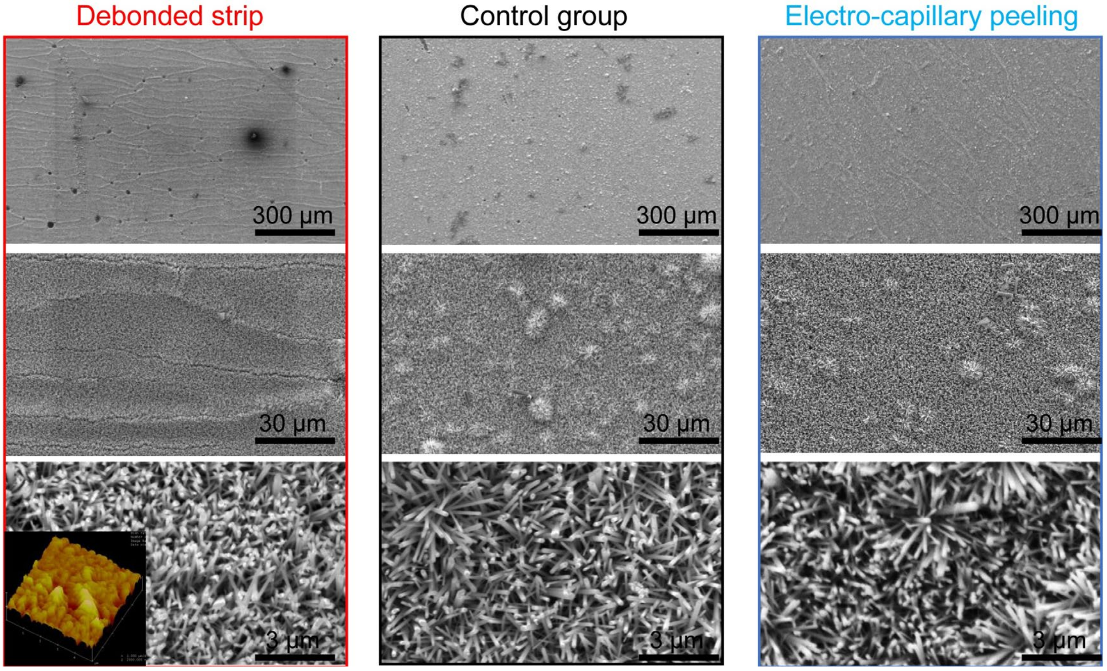

Supplementary Fig. 6. SEM and AFM tests of ZnO nanolayer on the film. Black, red, and blue boxes mark three groups of ZnO nanorods characterization. The control group is the ZnO nanolayer on the film without peeling. The insert photo is the AFM result of the crack on the ZnO nanolayer.

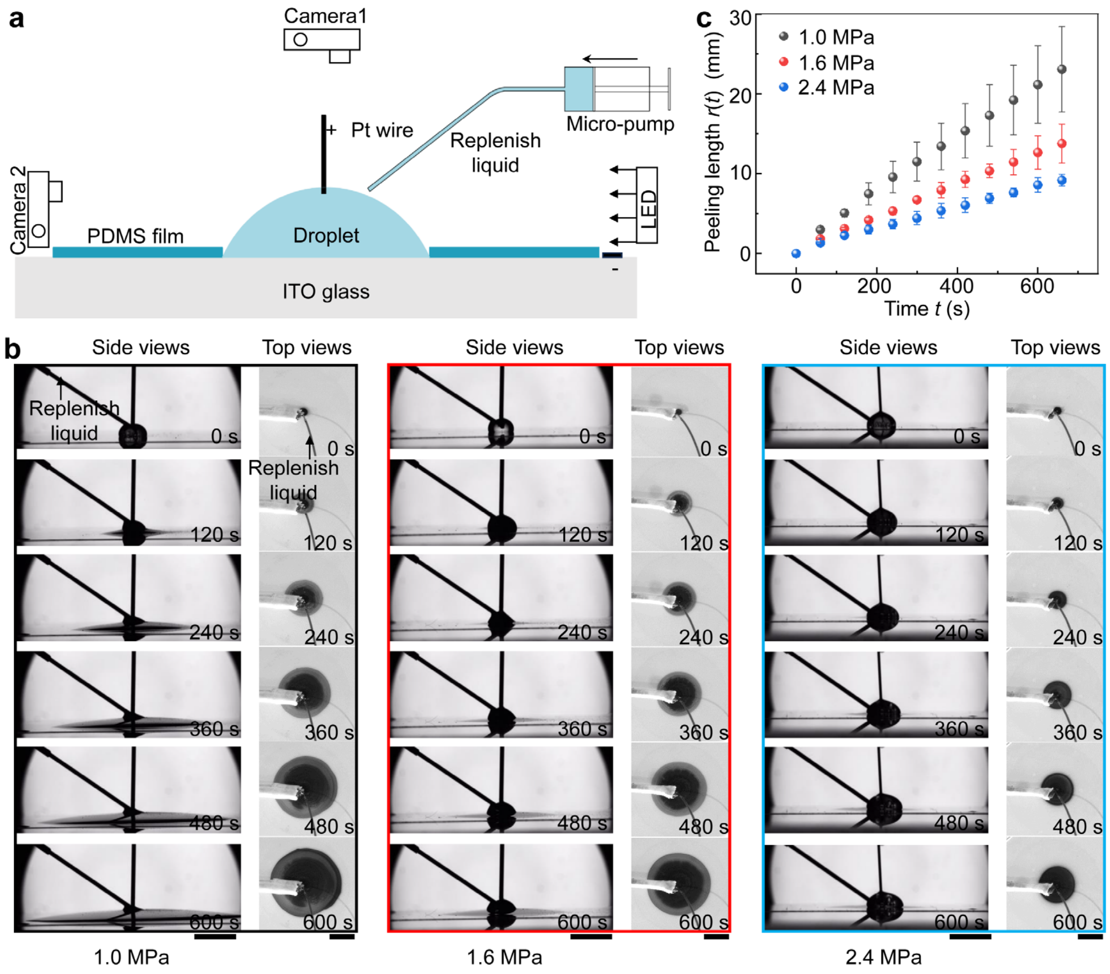

Supplementary Fig. 7. A long-term detachment strategy of the electro-capillary peeling method. a Schematic illustration of the long-term detachment strategy. A micropump is used to replenish fluid during the electro-capillary peeling. **b** Side and top views of the long-term peeling process. Black, red, and blue boxes are used to mark the thin film detachment with elastic moduli of 1.0, 1.6, and 2.4 MPa, respectively. The tested time is 660 s, and the scale bars in the side and top views are 1 cm. **c** Statistical results of peeling length in the long-term peeling. The thickness of tested films in **b** and **c** are 100 μm, and the error bars in **c** are the standard deviation of the raw data.

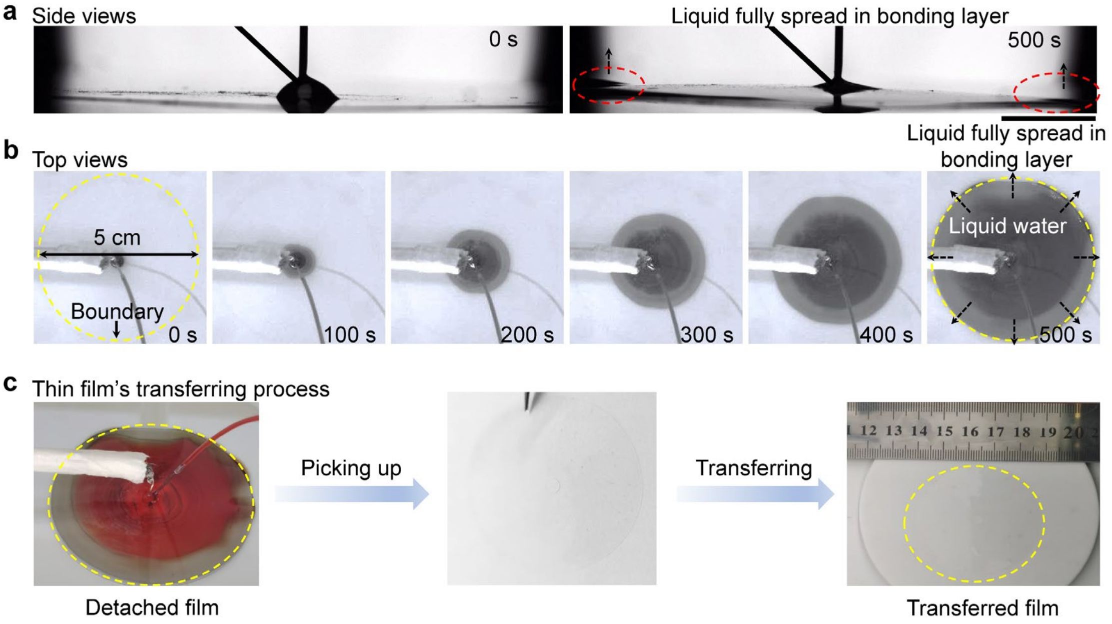

Supplementary Fig. 8. The entire electro-capillary peeling process of a thin film (5 cm in diameter). **a-b** Side and top views of the entire electro-capillary peeling process. When the liquid spreads in the bonding layer fully, it seeps from the thin film's boundary. Yellow and red dot lines mark the thin film's boundary and the exudated liquid water, respectively. **c** Thin film transfer process. After the film is entirely detached by the electro-capillary peeling method, it can be picked up and transferred to another substrate.

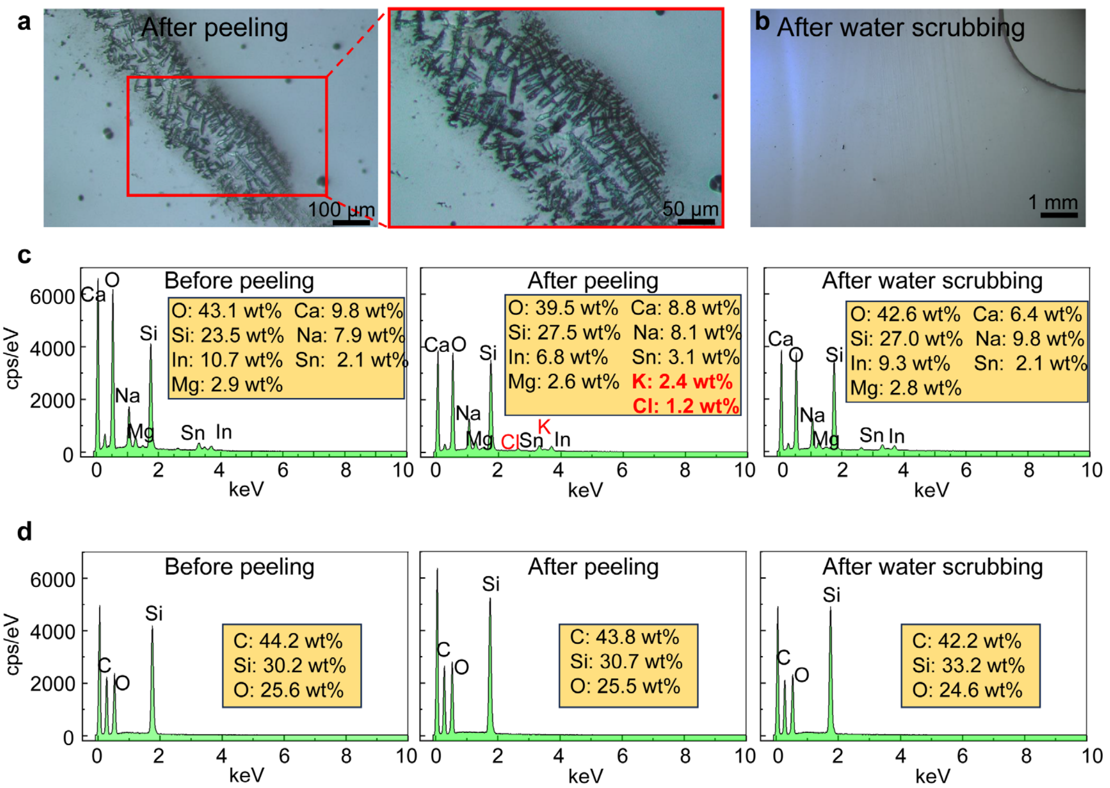

Supplementary Fig. 9. Photomicrograph and EDS characterization of the substrate/film after thin film detachment (*U* = 4.5 V). a Photomicrograph of the substrate after thin film detachment. The dendrite line represents the potassium chloride (KCl) precipitation on the substrate. **b** Optical image of the detached film on the substrate after water scrubbing. **c-d** EDS characterization of the substrate and the PDMS film, respectively. Some potassium chlorides (KCl) are deposited on the substrate (about 1.2 wt%) but not on the films. The EDS results show no discernible differences between the substrate/film before peeling and after the water scrubbing.

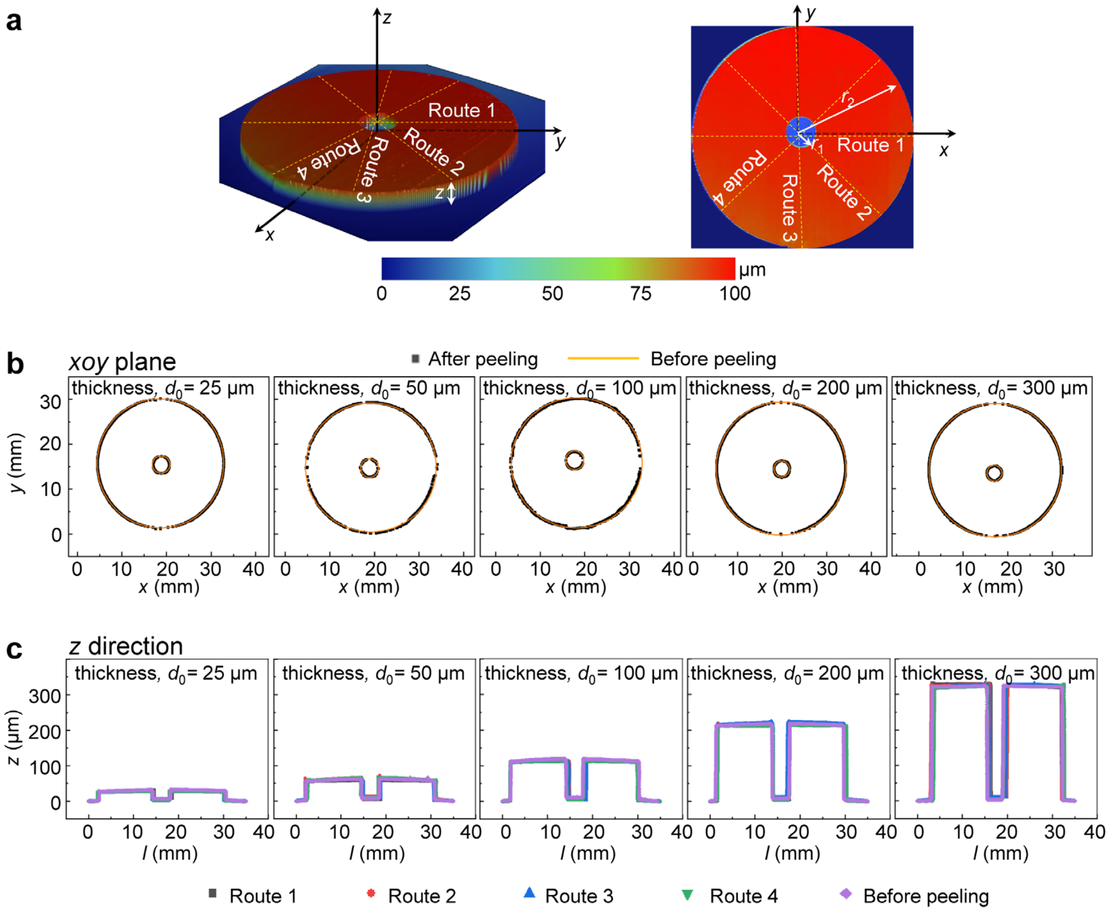

Supplementary Fig. 10. Deformation of detached films after transferring. a Side and top views of PDMS films' three-dimensional topography. r_1 and r_2 are the inner and outer boundaries' radius of the thin film, respectively, and routes 1, 2, 3, and 4 are the surface profiler scan paths. **b** The position of the PDMS film's inner and outer boundaries before and after transferring. Black square dots and the orange solid line represent the position of the boundaries after and before transferring, respectively. **c** The *z*-coordinate of PDMS films' thickness before and after transferring. *z* and *l* represent the *z*-coordinate and the scan length.

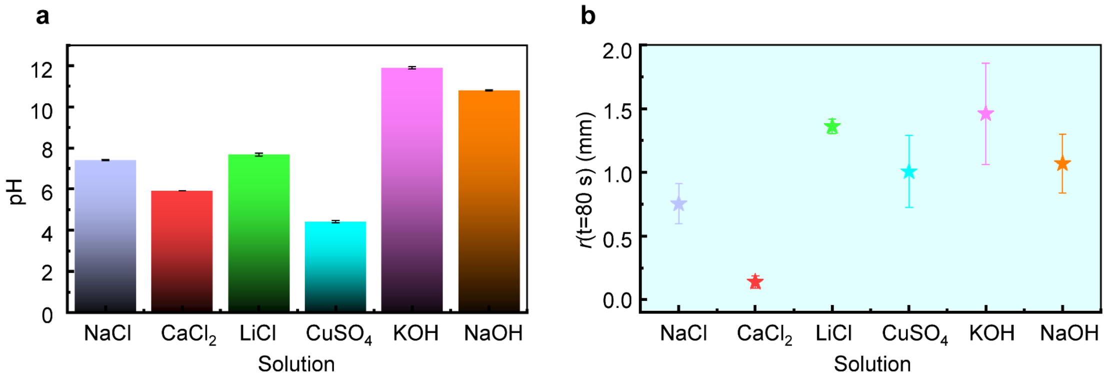

Supplementary Fig. 11. Characterization of pH and peeling length at different solutions. a The pH value of diverse solutions, including NaCl, $CaCl_2$, LiCl, $CuSO_4$, KOH, and NaOH solution. **b** The peeling length of electro-capillary peeling in various solutions, such as neutral, acidic, and alkaline solutions. The results are obtained with PDMS films of 100 μm at the voltage of 2.5 V. Although the electro-capillary peeling performance is affected by the types of solution, it is appropriate in broad ranges of solutions. The concentration of the tested solutions is 1.0 mol L^{-1}.

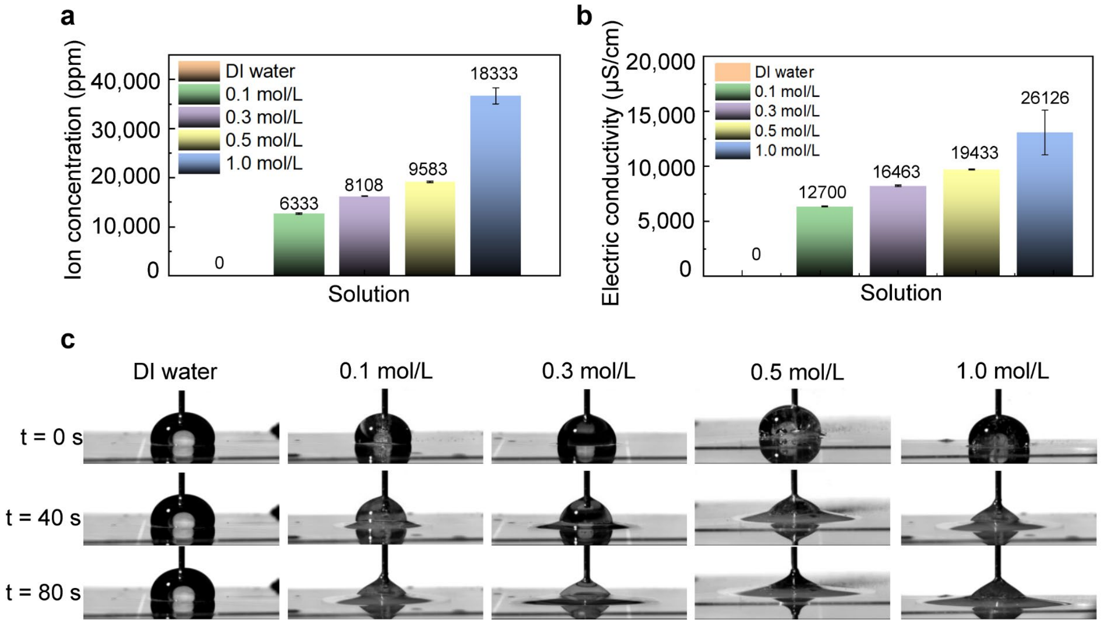

Supplementary Fig. 12. Electro-capillary peeling method with a droplet of different ion concentrations. a-b The ion concentration and electric conductivity of droplets at various concentrations of 0.1, 0.3, 0.5, and 1.0 mol L^{-1}. **c** The characterization of the electro-capillary peeling method with a droplet of ion concentrations from 0 to 1.0 mol L^{-1}. The tested voltage is 2.5 V.

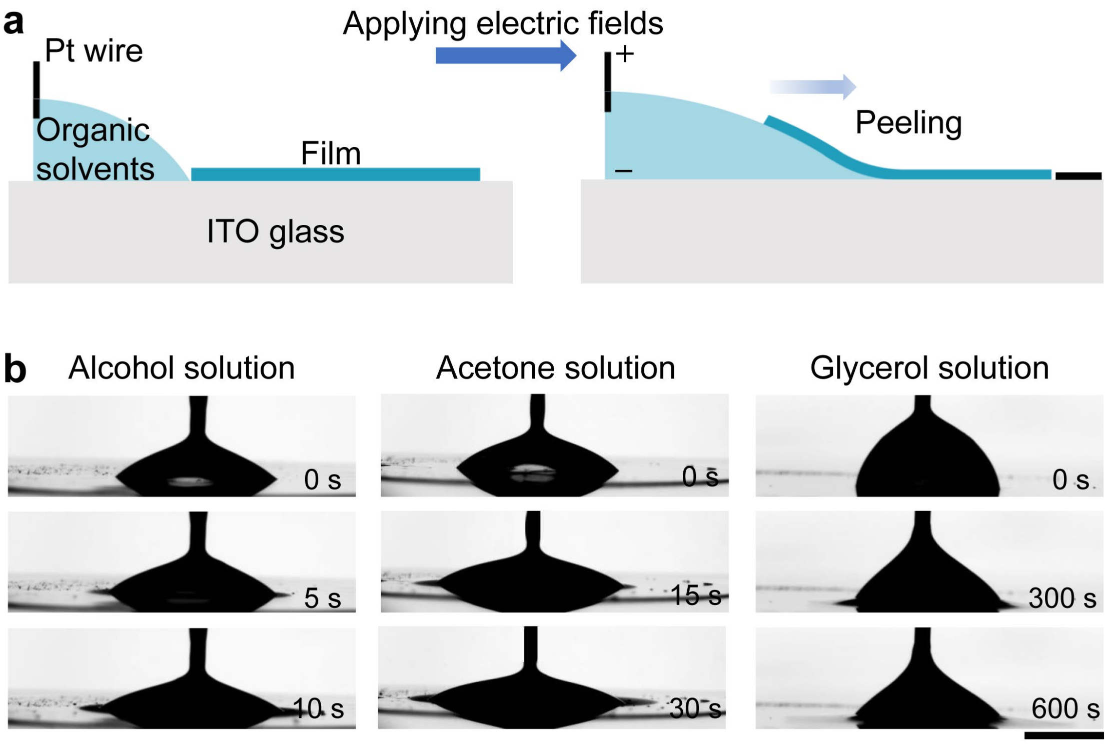

Supplementary Fig. 13. Electro-capillary peeling method using different types of organic solvents. a Schematic diagram of electro-capillary peeling applied with organic solvents. **b** The detaching process of the electro-capillary peeling method applied with organic solvents. The tested organic solvents are (±)-Camphor-10-sulfonic acid/alcohol, (±)-Camphor-10-sulfonic acid/acetone, and sodium hydroxide/glycerol solutions. The concentrations of solutions are 0.5 mol L^{-1}, and the scale bars are 2 mm.

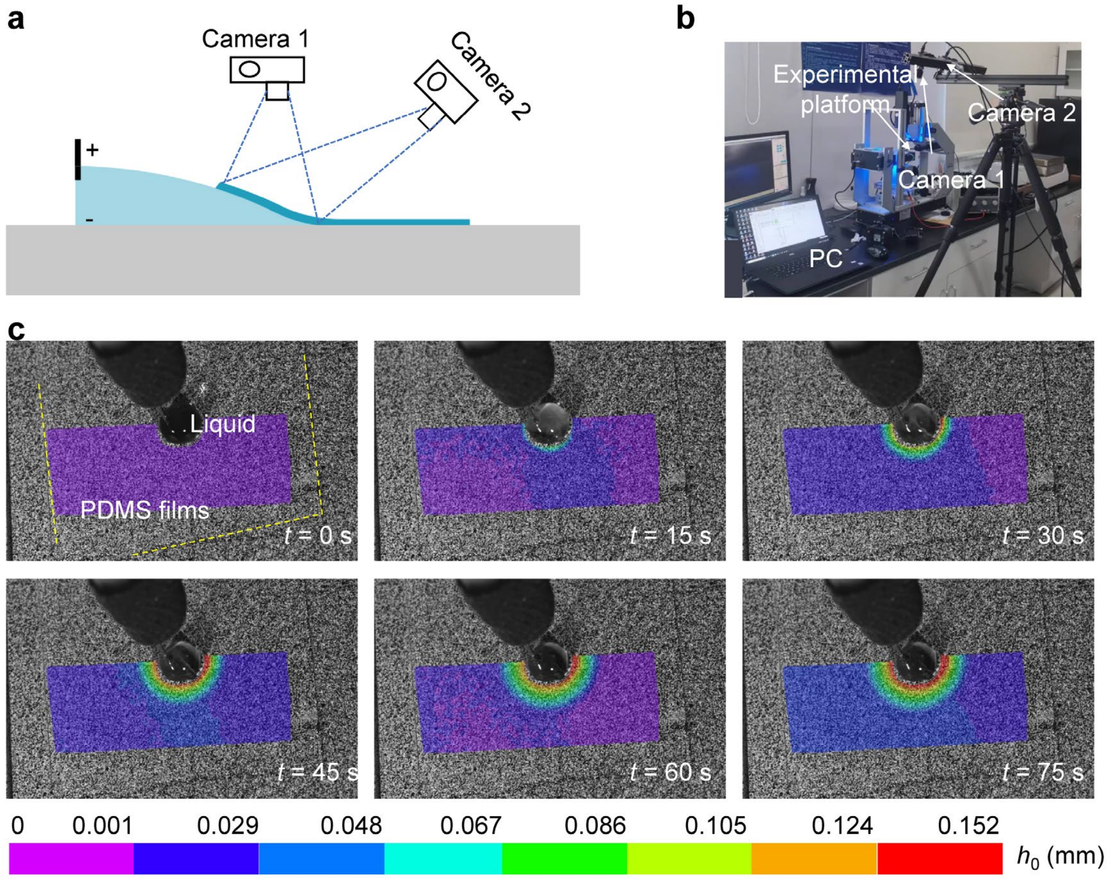

Supplementary Fig. 14. The experimental setup of 3D DIC and the deformation of film in the electro-capillary peeling method. a-b Experimental apparatus setup of 3D DIC. **c** Displacement fields of film during the electro-capillary peeling process. Yellow dotted lines are used to mark the boundary of PDMS film. Color bars represent the displacement value.

Supplementary Tables

Supplementary Table 1. The wet adhesion of PDMS films (1.6 MPa) with different thicknesses.

Thickness (μm)	Wet adhesion (mJ m^{-2})
25	130 ± 25
50	122 ± 38
100	142 ± 43
200	129 ± 66
300	146 ± 28

Supplementary Table 2. The wet adhesion of PDMS films (100 μm) with various elastic moduli.

Elastic modulus (MPa)	Wet adhesion (mJ m^{-2})
1.0	153 ± 40
1.6	142 ± 43
2.4	126 ± 23

Supplementary Table 3. The wet adhesion of PDMS films (100 μm) with various elastic moduli.

Types	Wet adhesion (mJ m^{-2})
PEN	37 ± 38
PET	72 ± 43
Hydrogel	3656 ± 772

The wet adhesion of hydrogel films here was characterized by a peeling test at the angle of 90° under water due to the hydrogel films being always destroyed in the water blister test.

Supplementary Table 4. The elastic modulus of functional films used in the experiment.

Types	Elastic modulus (MPa)
Hydrogel	0.1
PET	2600
PEN	3000
