# Supplementary material for: Electro-capillary peeling of thin films
Source: Nat Commun. 2023 Oct 3;14:6150. doi: 10.1038/s41467-023-41922-2 (PMC10547721; doi:10.1038/s41467-023-41922-2)
Supplement: Supplementary file 3 — Description of Additional Supplementary Files [file 41467_2023_41922_MOESM3_ESM.docx]

**Description of Additional Supplementary Files**

**File Name: Supplementary Movie 1**

**Description:** Side and top views of electro-capillary peeling for detaching PDMS films on the ITO glass. The PDMS film with a thickness of 100 μm and an elastic modulus of 2.4 MPa is detached by the electro-capillary peeling method at a voltage of 1.5 V.

**File Name: Supplementary Movie 2**

**Description:** Side and top views of electro-capillary peeling at various applied voltages. The applied voltages are 1.5, 2.5, 3.5, and 4.5 V. The thickness and elastic modulus of PDMS film are 100 μm and 2.4 MPa, respectively.

**File Name: Supplementary Movie 3**

**Description:** Planar peeling mode of electro-capillary peeling method. The applied voltage is 1.5 V, and the thickness and elastic modulus of PDMS film are 100 μm and 2.4 MPa, respectively.

**File Name: Supplementary Movie 4**

**Description:** Displacement and strain fields of PDMS films characterized by 3D DIC method. Colour bars represent the displacement and strain values. The applied voltage is 2.5 V, and the thickness and elastic modulus of PDMS film are 100 μm and 2.4 MPa, respectively.
